# Comparison of qSOFA Score and NEWS for Predicting Hospital Admission and Mortality in the Emergency Department: A Prospective Observational Study

**DOI:** 10.3390/healthcare14203521

**Published:** 2026-10-19

**Authors:** Abdurrahman Yılmaz, Ali Ünal

**Affiliations:** Department of Emergency Medicine, Uşak Training and Research Hospital, Uşak University Faculty of Medicine, Uşak 64300, Türkiye; ali.unal@usak.edu.tr

**Keywords:** NEWS, qSOFA, emergency department, triage, mortality, hospital admission, early warning score

## Abstract

**Background/Objectives:** This study compared the performance of the National Early Warning Score (NEWS) and the quick Sequential Organ Failure Assessment (qSOFA) score for predicting hospital admission and in-hospital mortality among adult patients triaged to the high-acuity area of the emergency department (ED). qSOFA was derived and validated in patients with suspected infection, and only 8.1% of this cohort carried a preliminary diagnosis of sepsis; its evaluation here is therefore exploratory. **Methods:** A total of 270 patients were included in this prospective observational study. Vital signs, Glasgow Coma Scale scores, and both scores were recorded at ED presentation. Discrimination was assessed by receiver operating characteristic (ROC) analysis, with the two scores compared using the DeLong test. Sensitivity and specificity were then examined separately at both conventional and data-derived thresholds. Bootstrap internal validation, calibration plots, Brier scores and decision-curve analysis were also performed, all computed using the development dataset. **Results:** Of 268 patients evaluable for the admission outcome (two ED deaths were excluded as a competing outcome), 35.8% (*n* = 96) were admitted, and 7.0% (*n* = 19 of 270) died in the hospital. No statistically significant difference was found between the AUCs of the two scores for admission (0.709 vs. 0.695; *p* = 0.627) or mortality (0.900 vs. 0.862; *p* = 0.150). At the conventional qSOFA score threshold of ≥2, the expected high-specificity, low-sensitivity profile was reproduced for mortality (96.8% and 52.6%), whereas the cohort-derived qSOFA score threshold of ≥1 favored sensitivity (84.2%). Bootstrap correction showed modest optimism. Calibration assessment was limited by the small number of events, and both score-specific models showed apparent net benefit over treat-all and treat-none strategies across most thresholds examined. Each score was independently associated with mortality in exploratory score-specific models. **Conclusions:** NEWS and qSOFA showed no statistically significant difference in discrimination in this high-acuity ED cohort. With only 19 deaths, the mortality analyses and the cohort-specific thresholds are exploratory and require confirmation in larger, multicenter cohorts before any clinical use.

## 1. Introduction

Early identification of patients at risk of clinical deterioration is a central challenge in emergency medicine. Emergency departments (EDs) receive a heterogeneous population ranging from stable, low-acuity presentations to critically ill patients requiring immediate intervention, and the ability to rapidly distinguish between these groups directly influences resource allocation, disposition decisions, and clinical outcomes.

A variety of bedside scoring systems have been developed to support this triage process. The National Early Warning Score (NEWS), introduced by the Royal College of Physicians, integrates seven physiological parameters—respiratory rate, oxygen saturation, supplemental oxygen use, temperature, systolic blood pressure, heart rate, and level of consciousness—into a single composite score originally intended to detect clinical deterioration in hospitalized patients and subsequently adopted for use in the ED setting [1]. In parallel, the Third International Consensus Definitions for Sepsis and Septic Shock (Sepsis-3) introduced the quick Sequential Organ Failure Assessment (qSOFA) score, a simplified three-parameter tool (systolic blood pressure, respiratory rate, and altered mentation) intended to identify patients with suspected infection who are at increased risk of poor outcomes outside the intensive care unit [2]. It should be noted that qSOFA was specifically derived and validated in patients with suspected infection, whereas NEWS was designed as a general-purpose deterioration score independent of etiology; this difference in intended scope is relevant when interpreting comparative studies, including the present one.

Since their introduction, NEWS and qSOFA have been extensively compared across diverse ED populations, including patients with suspected sepsis, COVID-19, and immunocompromising conditions. While several large cohort studies have reported superior discriminative performance for NEWS—attributed to its broader physiological parameter set and higher sensitivity—other studies have found no statistically significant difference between the two scores, and in some subpopulations, particularly among elderly patients, qSOFA has outperformed NEWS. This heterogeneity in the literature underscores the need for further comparative data across different clinical settings and patient populations.

In this study, we compared the discriminative performance of NEWS and qSOFA for in-hospital mortality, ICU admission and hospital admission among adult patients triaged to the high-acuity area of the emergency department. Unlike most prior comparative studies, our cohort was not restricted to patients with suspected infection: only 8.1% carried a preliminary diagnosis of sepsis. qSOFA was therefore evaluated as a generic physiological risk marker rather than according to its originally intended clinical purpose, and this evaluation is exploratory throughout.

## 2. Materials and Methods

### 2.1. Study Design and Setting

This was a prospective observational study conducted in the Emergency Department of Uşak Training and Research Hospital, Uşak, Türkiye. Patients were consecutively enrolled between December 2025 and March 2026. Data were collected prospectively at the time of patient presentation, prior to the initiation of any treatment.

No formal a priori sample size or power calculation was performed. All consecutive adult patients meeting the inclusion criteria during the study period were enrolled, and the final sample size (*n* = 270) was therefore determined pragmatically by the study duration rather than by a prespecified statistical target; this is noted as a limitation in Section 5.

### 2.2. Study Population

Patients evaluated in the high-acuity (“red”) triage area of the ED, in accordance with the Turkish Ministry of Health emergency department triage guidelines, were included in this study. Under this national system, patients are assigned at first contact by a triage nurse, with emergency physician oversight, to a red (high-acuity), yellow (intermediate) or green (low-acuity) area. Red-area criteria are applied on the basis of the presenting complaint together with initial vital signs and level of consciousness and cover patients with altered consciousness, respiratory distress, hemodynamic instability, active bleeding, suspected acute coronary syndrome, stroke or sepsis, and any presentation judged to carry a risk of imminent deterioration. Because these criteria are applied before diagnostic evaluation, the red area is a risk-capture category rather than a retrospective severity classification and does not correspond precisely to the resuscitation or trauma bay areas of some international triage systems. It therefore accommodates a wide spectrum of illness severity, from patients ultimately discharged to those requiring intensive care (Table 1).

The inclusion criterion was defined as adult patients evaluated in the high-acuity triage area at the time of ED presentation. Patients presenting due to trauma, those brought to the ED in cardiac arrest, patients with incomplete vital signs at presentation, pregnant patients, and patients who refused treatment were excluded from the study.

With this approach, the study population was defined as adult patients considered by ED triage staff to require high-acuity assessment, encompassing a range of illness severity from patients ultimately discharged to those requiring intensive care (Table 1).

### 2.3. Data Collection and Outcomes

Demographic and clinical data of patients were recorded prospectively at the time of ED presentation. Data collected included age, sex, presence of comorbid disease, vital signs (pulse rate, respiratory rate, systolic and diastolic blood pressure, body temperature, and oxygen saturation), level of consciousness (recorded using both the Glasgow Coma Scale [GCS] and the AVPU [Alert, Voice, Pain, Unresponsive] scale), and supplemental oxygen status. The AVPU scale was used to calculate the consciousness component of NEWS, in accordance with the original NEWS algorithm, whereas altered mentation for qSOFA was defined as GCS < 15, in accordance with the original Sepsis-3 definition of qSOFA.

Using these parameters, the National Early Warning Score (NEWS) and quick Sequential Organ Failure Assessment (qSOFA) scores were calculated during the triage assessment of each patient. All measurements were obtained during the patient’s initial assessment in the ED, prior to the initiation of any treatment. NEWS and qSOFA scores were calculated for research purposes and were not made available to treating physicians, who were therefore blinded to these scores during clinical decision-making. The original NEWS algorithm (Royal College of Physicians, 2012) [1] was used throughout; the 2017 NEWS2 revision was not applied.

The outcomes of the study were hospital admission and in-hospital mortality. For reporting and interpretation, these are treated as a hierarchy: in-hospital mortality is the principal outcome, ICU admission is the secondary outcome of greatest clinical interest, and the composite hospital admission endpoint is an exploratory outcome because the decision to admit is strongly influenced by physician judgment, bed availability, local practice and institutional policy rather than by physiology alone. Hospital admission was defined as admission to either the ward or the intensive care unit (*n* = 96 of 268 evaluable patients). The two patients who died in the ED prior to a ward or ICU bed being assigned were excluded from admission-specific analyses (yielding *n* = 268 for this outcome) rather than classified as “not admitted,” because death in the ED represents a competing outcome that is not comparable to either successful discharge or ward/ICU admission; these two patients are instead captured under the mortality outcome, for which the full cohort (*n* = 270) was retained. In-hospital mortality was defined as all-cause death occurring during the index hospital episode, including death in the ED, and 19 of the 270 patients (7.0%) met this definition. One further patient, discharged from the ED and never admitted, was subsequently recorded as deceased on day 10; because no in-hospital episode occurred, this patient did not meet the outcome definition and was retained in the cohort as a non-event, with the death reported descriptively in Section 3. This death came to light through a notification recorded in our hospital information system; no systematic search of national death records was performed for the cohort. Outcome ascertainment was otherwise confined to our own institution: patients discharged from the ED were not followed prospectively, and deaths, readmissions or presentations at other institutions were not systematically sought. An unplanned return to the same ED within 24 h was also recorded for discharged patients; returns to other hospitals were likewise not captured.

Comorbid disease was recorded using a predefined list of ten categories (hypertension, diabetes mellitus, coronary artery disease, heart failure, chronic obstructive pulmonary disease, chronic kidney disease, cerebrovascular disease, malignancy, chronic liver disease and immunosuppression) together with an “other documented chronic disease” category. The binary covariate “comorbid disease present,” used in all regression models, denotes at least one such condition; patients could carry more than one, and 48.3% of those with any comorbidity did so.

### 2.4. Statistical Analysis

Data were analyzed using jamovi statistical software (Version 2.7; the jamovi project, 2025). The distribution of the data was assessed using the Kolmogorov–Smirnov test. Continuous variables that did not follow a normal distribution were presented as median (25th–75th percentile), and categorical variables were expressed as numbers and percentages [*n* (%)].

The Mann–Whitney U test was used to compare continuous variables between two groups, and the chi-square test, with Yates’ continuity correction for 2 × 2 tables, was used to compare categorical variables.

The diagnostic performance of NEWS and qSOFA scores in predicting hospital admission and mortality was evaluated using receiver operating characteristic (ROC) curve analysis. The area under the curve (AUC) and 95% confidence intervals were calculated. Optimal cutoff points were determined using the Youden index. Differences between the AUC values of the scores were compared using the DeLong test. In addition to the data-derived optimal cutoffs, the conventional thresholds used in clinical practice and in most of the comparative literature (NEWS ≥ 5 and NEWS ≥ 7; qSOFA ≥ 2) were evaluated for both outcomes. Because the Youden-derived thresholds were both selected and evaluated in the same dataset, they were subjected to internal validation by bootstrap resampling (2000 resamples; Harrell’s optimism correction), the Youden-optimal threshold being re-selected within each resample and then evaluated in the original cohort.

Multivariable logistic regression analysis was performed to identify independent predictors associated with hospital admission and mortality. Results were reported as odds ratios (ORs) with 95% confidence intervals.

Because NEWS and qSOFA share overlapping physiological components (respiratory rate, systolic blood pressure, and level of consciousness) and were entered simultaneously alongside supplemental oxygen and GCS in the initial multivariable models, potential multicollinearity was addressed with two additional, score-specific parsimonious models: Model A (NEWS, age, and comorbid disease) and Model B (qSOFA, age, and comorbid disease). Given the limited number of mortality events (*n* = 19), Firth’s penalized (bias-reduced) logistic regression was performed as a post hoc sensitivity analysis for all mortality models, undertaken to address concerns about overfitting given the limited number of mortality events, and model fit was compared using the Akaike Information Criterion (AIC). As a secondary analysis, intensive care unit (ICU) admission and ward admission were also examined as separate outcomes, in addition to the composite hospital admission outcome, and positive and negative predictive values (PPV and NPV) and likelihood ratios (LR+, LR−) were calculated at the previously determined optimal cutoff values. Collinearity between NEWS and qSOFA was assessed using the Pearson correlation coefficient and variance inflation factors (VIFs) calculated for the full six-variable model. Model calibration for the primary score-specific models (Model A and Model B) was assessed using the Hosmer–Lemeshow goodness-of-fit test. Because only 19 deaths occurred, all mortality regression analyses are exploratory. The two score-specific models were constructed on clinical grounds—each score with age and comorbid disease—and are used as the basis for interpretation, with the full six-variable model retained as a supporting comparison.

The following analyses, together with the conventional thresholds and bootstrap validation described above, were added during revision in response to peer review rather than planned in advance; this applies to the outcome hierarchy and the choice of score-specific models as the interpretive basis as well. The score-specific models were internally validated using 2000 bootstrap resamples, the model being refitted within each resample and evaluated in the original cohort, yielding optimism-corrected AUCs, Brier scores and calibration slopes. Calibration was displayed graphically as observed versus predicted probability (deciles for hospital admission; quintiles for mortality) alongside the Hosmer–Lemeshow test. Decision-curve analysis was used to estimate net benefit relative to treat-all and treat-none strategies. All predicted probabilities used for calibration, Brier scores and decision curves were generated from the standard (unpenalized) logistic models; Firth’s penalized estimates are reported only as a sensitivity analysis of the regression coefficients. The calibration plots and decision curves show apparent performance in the development dataset and are not optimism-corrected. Finally, a subgroup analysis restricted to the 22 patients carrying a preliminary diagnosis of sepsis was performed and is reported descriptively.

A *p*-value < 0.05 was considered statistically significant in all analyses.

## 3. Results

A total of 270 patients were included in the study. Of the patients, 47.8% (*n* = 129) were female and 52.2% (*n* = 141) were male.

Regarding the mode of presentation, 54.1% (*n* = 146) of patients presented on their own, 40.4% (*n* = 109) via the 112 emergency medical services, and 5.6% (*n* = 15) by referral from another facility.

Regarding comorbidity distribution, 34.1% (*n* = 92) of patients had no comorbid disease, while 65.9% (*n* = 178) had at least one comorbid condition. Counting every documented condition, the most frequent comorbidities were hypertension (42.2%; *n* = 114), diabetes mellitus (30.7%; *n* = 83), coronary artery disease (15.9%; *n* = 43), and chronic obstructive pulmonary disease (9.3%; *n* = 25); 48.3% of patients with any comorbidity carried more than one.

The most common presenting complaints were dyspnea (22.2%; *n* = 60) and general clinical deterioration (21.5%; *n* = 58), followed by palpitations (8.5%; *n* = 23), nausea/vomiting (8.1%; *n* = 22), abdominal pain (6.7%; *n* = 18), chest pain (6.3%; *n* = 17), and cerebrovascular events (6.3%; *n* = 17).

A preliminary diagnosis of sepsis was absent in 91.9% (*n* = 248) of patients and present in 8.1% (*n* = 22). Supplemental oxygen use was recorded in 21.9% (*n* = 59) of patients; no supplemental oxygen use was recorded in the remaining 78.1% (*n* = 211). According to the AVPU assessment, 94.1% (*n* = 254) of patients were alert.

Regarding ED disposition, 63.7% of patients (*n* = 172) were discharged, 22.6% (*n* = 61) were admitted to the ward, 13.0% (*n* = 35) were admitted to the intensive care unit, and 0.7% (*n* = 2) died in the ED. The in-hospital mortality rate was 7.0% (*n* = 19 of 270). Because death in the ED represents a competing outcome rather than a failure to be admitted, these two patients were excluded from the hospital admission analyses reported below (*n* = 268 evaluable), while remaining included in the mortality analyses (*n* = 270).

Of the 172 patients discharged directly from the ED, median NEWS was 1.5 (IQR: 1–3) compared with 4.0 (2–7) in those admitted; 83.7% (*n* = 144) had a NEWS within the low-risk band (0–4), and 90.1% (*n* = 155) had a qSOFA score of 0. Three discharged patients (1.7%) made an unplanned return to the same ED within 24 h. One discharged patient—an 83-year-old with a NEWS of 8 and a qSOFA score of 2—died on day 10 without having been admitted to our institution; as set out in Section 2.3, this death did not meet the in-hospital mortality definition, and the patient was counted as a non-event. Because discharged patients were not followed prospectively and presentations to other institutions were not systematically sought, these figures are a minimum estimate of adverse events after discharge and are descriptive rather than evidence that discharge was safe.

Regarding demographic and clinical parameters, the median age of patients was 66.0 (IQR: 47.3–77.0) years. Median pulse rate was 90 (76.3–106) bpm, respiratory rate was 18 (17–20) breaths/min, systolic blood pressure was 130 mmHg (110–156), and diastolic blood pressure was 75 mmHg (64–86). Median body temperature was 36.4 °C (36.2–36.7), and oxygen saturation was 97% (94–98). The median Glasgow Coma Scale score was 15 (15–15), the median qSOFA was 0 (0–0), and the median total NEWS was 2 (1–5). Mean arterial pressure was 96.0 ± 20.5 mmHg. Among the 19 patients who died in hospital, the date of death was recorded for 18; in these patients, the mean time from ED presentation to death was 4.61 ± 3.52 days (Table 1).

When discharged patients were compared with those admitted to the ward or ICU (the two patients who died in the ED were excluded from this comparison; see Section 2.3), the total NEWS was significantly higher in admitted patients (median: 4.0 vs. 1.5; *p* < 0.001). Similarly, the qSOFA score was significantly higher in admitted patients (*p* < 0.001). Admitted patients were older (73.0 vs. 57.5; *p* < 0.001), had comorbidities more frequently (82.3% vs. 56.4%; *p* < 0.001), and had supplemental oxygen use recorded more often (35.4% vs. 13.4%; *p* < 0.001). GCS values also differed significantly between groups (*p* < 0.001), although the median GCS was 15 in both groups (Table 2).

When patients who died in hospital were compared with survivors, the total NEWS was significantly higher in those who died (median: 10.0 vs. 2.0; *p* < 0.001). The qSOFA score was also significantly higher (*p* < 0.001). Patients who died were older (82.0 vs. 64.0; *p* < 0.001), had supplemental oxygen use recorded more frequently (73.7% vs. 17.9%; *p* < 0.001), and had lower GCS values (*p* < 0.001); the difference in comorbidity prevalence was not statistically significant (84.2% vs. 64.5%; *p* = 0.135) (Table 3).

In the ROC analysis for predicting hospital admission (*n* = 268; ward or ICU admission, *n* = 96), the AUC for the total NEWS was 0.709 (CI: 0.641–0.777; *p* < 0.001), and for qSOFA it was 0.695 (CI: 0.639–0.750; *p* < 0.001). No statistically significant difference was found between the AUC values of the two scores (*p* = 0.627) (Figure 1).

The optimal cutoff for NEWS was ≥3, with a sensitivity of 70.8% and specificity of 69.2%, while for qSOFA the optimal cutoff was ≥1, with a sensitivity of 47.9% and specificity of 90.1%.

In the ROC analysis for predicting in-hospital mortality, the AUC for the total NEWS was 0.900 (CI: 0.814–0.985; *p* < 0.001), and for qSOFA it was 0.862 (CI: 0.767–0.958; *p* < 0.001) (Figure 2). No statistically significant difference was found between the AUC values of the two scores (DeLong *p* = 0.150) (Table 4). For NEWS, a cutoff of ≥7 yielded a sensitivity of 78.9% and specificity of 90.4%; for qSOFA, a cutoff of ≥1 yielded a sensitivity of 84.2% and specificity of 80.5%.

To further characterize the predictive performance of the optimal cutoffs identified above, positive and negative predictive values and likelihood ratios were calculated (Table 5). Given the low prevalence of in-hospital mortality (7.0%) in this cohort, NPV was uniformly high (≥98%) for both scores at their respective mortality cutoffs, whereas PPV was modest, as expected for a low-prevalence outcome.

Because the thresholds above were derived within this cohort, the conventional thresholds used in routine practice were also evaluated (Table 5). For mortality, qSOFA ≥ 2 reproduced the high-specificity, low-sensitivity profile reported in the sepsis literature (sensitivity 52.6%, specificity 96.8%, LR+ 16.5), in contrast to the cohort-derived qSOFA ≥ 1 threshold, which favored sensitivity (84.2%) at the cost of specificity (80.5%). NEWS ≥ 5 was highly sensitive (89.5%) but less specific (76.9%), whereas NEWS ≥ 7—the conventional high-risk threshold, which coincided with the Youden-derived cutoff here—gave a more balanced profile (78.9% and 90.4%). For hospital admission, qSOFA ≥ 2 was highly specific (98.8%) but identified only 14.6% of admitted patients. These findings illustrate the influence of threshold selection on the observed sensitivity–specificity profile.

Because the composite hospital admission outcome combined ward and ICU admission, and because this composite endpoint is influenced by non-physiological factors such as bed availability and clinician judgment, ICU admission and ward admission were additionally examined as separate outcomes (Table 6). For ICU admission (*n* = 35), the AUC was 0.728 (95% CI: 0.638–0.819) for qSOFA and 0.710 (0.604–0.816) for NEWS; for ward admission (*n* = 61), it was 0.638 (0.558–0.718) for NEWS and 0.607 (0.540–0.674) for qSOFA. Neither between-score difference was statistically significant (DeLong *p* = 0.634 and 0.393).

In the full six-variable multivariable logistic regression model, NEWS (OR: 1.19; 95% CI: 1.01–1.41; *p* = 0.039), qSOFA (OR: 2.67; 95% CI: 1.23–5.77; *p* = 0.013), and comorbid disease (OR: 2.45; 95% CI: 1.12–5.32; *p* = 0.024) were all independent predictors of hospital admission (Table 7).

Because NEWS and qSOFA were entered simultaneously in the model above alongside variables that directly or partially overlap with their own components (supplemental oxygen and GCS), two additional score-specific models were fitted for hospital admission: Model A (NEWS, age and comorbid disease) and Model B (qSOFA, age and comorbid disease) (Table 8). In both, each score was independently associated with admission when modeled without the other (NEWS: OR 1.29, *p* < 0.001; qSOFA: OR 4.85, *p* < 0.001), with AIC 298.8, 288.7 and 288.5 for Model A, Model B and the full model respectively.

In the multivariable analysis for predicting in-hospital mortality, NEWS was an independent predictor (OR: 1.73; 95% CI: 1.21–2.47; *p* = 0.003), whereas qSOFA was not (OR: 1.40; 95% CI: 0.39–4.99; *p* = 0.608). Age (OR: 1.10; 95% CI: 1.03–1.18; *p* = 0.005) and GCS (OR: 0.69; 95% CI: 0.48–0.98; *p* = 0.040) were also independently associated with mortality (Table 9).

Given the well-documented concern that entering NEWS and qSOFA in the same multivariable model—alongside supplemental oxygen and GCS, which are direct or overlapping components of these scores—can obscure each score’s independent contribution through multicollinearity, and given the limited number of mortality events relative to the six covariates in the full model (Table 9), two parsimonious, score-specific models were fitted for mortality: Model A (NEWS, age, and comorbid disease) and Model B (qSOFA, age, and comorbid disease), each also re-estimated using Firth’s penalized logistic regression as a post hoc sensitivity analysis for rare events (Table 10).

In Model A, NEWS was independently associated with mortality (OR 1.65, 95% CI: 1.36–2.01, *p* < 0.001; Firth: OR 1.60). In Model B, qSOFA—not an independent predictor in the full six-variable model (*p* = 0.608)—was independently associated with mortality when modeled without NEWS, supplemental oxygen and GCS (OR 8.30, 95% CI: 3.67–18.78, *p* < 0.001; Firth: OR 7.40, 95% CI: 3.44–15.89). AIC was lowest for the full model (77.7), followed by Model A (81.5) and Model B (87.8), but AIC does not establish validity and must be weighed against the full model’s events-per-variable ratio of 3.2 (versus 6.3). Firth’s correction applied to the full model gave similar estimates for NEWS (OR 1.60, *p* = 0.004) and qSOFA (OR 1.42, *p* = 0.564), but GCS was significant in the standard model and not in the penalized one (*p* = 0.040 and 0.057); the correction therefore did alter the inferential status of one covariate, and we do not read the similarity of the remaining coefficients as evidence that the full model is stable.

Calibration was first assessed using the Hosmer–Lemeshow test. For hospital admission, no evidence of poor calibration was observed for either Model A (NEWS-based) or Model B (qSOFA-based) (χ^2^ = 7.85, df = 8, *p* = 0.448 and χ^2^ = 8.96, df = 8, *p* = 0.346), with adequate expected event counts per decile (all > 2). For mortality, the test was also non-significant (χ^2^ = 0.96 and 1.49, df = 8, *p* = 0.998 and 0.993), but with 19 deaths the expected event counts per decile were frequently below 1, so the test is uninformative here and is reported for transparency only. Calibration was therefore additionally assessed graphically and by Brier score and calibration slope, as reported below.

The number of patients and observed in-hospital deaths at each qSOFA value are shown in Table 11. Observed mortality rose across qSOFA categories, from 1.5% at qSOFA = 0 to 12.8% at 1 and 52.9% at 2, with the single patient scoring 3 having died. The upper categories are very sparse (*n* = 17 and *n* = 1), and the odds ratio estimated from them is correspondingly unstable; these counts are presented so that readers can judge the estimate directly.

Bootstrap internal validation indicated modest optimism, both in the data-derived thresholds and, separately, in the score-specific models. For the thresholds, the sensitivity of NEWS ≥ 7 fell from 78.9% to an optimism-corrected 74.7% and that of qSOFA ≥ 1 from 84.2% to 82.6%, with specificity essentially unchanged; for hospital admission, the corrected estimates were within 0.4 percentage points of the apparent values. For the score-specific models, which were validated separately, optimism-corrected AUCs were 0.936 (Model A) and 0.920 (Model B) for mortality and 0.748 and 0.774 for admission, with calibration slopes of 0.87–0.96 (Table 12). These corrections are estimated from the same 270 patients and, with 19 deaths, are imprecise: they indicate that the discrimination estimates are not grossly optimistic but do not exclude overfitting.

Grouped calibration plots are shown in Figure 3. For mortality, observed and predicted risks lay close to the diagonal across the five groups; for hospital admission, agreement was reasonable at low and intermediate predicted risk, with visible departures in the highest-risk deciles. These are apparent calibration plots computed in the development dataset, based on a few grouped points and, for mortality, on 19 events, and are shown without confidence intervals; they should not be taken as establishing good calibration. Brier scores—which summarize overall predictive performance rather than calibration alone—were 0.041 and 0.044 for mortality (optimism-corrected 0.045 and 0.048) and 0.186 and 0.177 for admission (corrected 0.192 and 0.182). Decision-curve analysis (Figure 4) describes the consequences of using each model to decide which patients warrant escalated assessment and early senior review; thresholds were examined from 1% to 40% for mortality and 1% to 80% for admission, ranges spanning plausible clinical attitudes to each decision given event rates of 7.0% and 35.8%. For mortality, both models gave positive net benefit throughout, with the NEWS-based model marginally higher than the qSOFA-based model (for example, 0.036 vs. 0.029 at a threshold of 0.20). For admission, the two were closely comparable, except that the qSOFA-based model fell marginally below treat-none between thresholds of approximately 0.73 and 0.78 (minimum net benefit −0.021). These curves were computed without optimism correction; the differences between models are small and were not formally tested, and they are descriptive only.

In the subgroup of 22 patients carrying a preliminary diagnosis of sepsis, in-hospital mortality was 31.8% (*n* = 7), median NEWS was 6.0 (IQR: 4.2–8.8), and median qSOFA was 1.0 (0–2). Within this subgroup the AUC for mortality was 0.924 (95% CI: 0.793–1.000) for NEWS and 0.800 (0.634–0.966) for qSOFA (DeLong *p* = 0.129); in the remaining 248 patients, among whom there were 12 deaths (4.8%), the corresponding values were 0.861 (0.736–0.986) and 0.817 (0.676–0.959). With seven deaths in the subgroup, these estimates are highly imprecise, and the two groups cannot be meaningfully compared. The recorded variable is, moreover, the preliminary diagnosis of sepsis rather than suspicion of infection of any kind, so patients with infections not considered septic at presentation fall into the larger group, and this analysis does not identify all patients in whom qSOFA would conventionally be applied.

Admission analyses are based on *n* = 268; the two patients who died in the ED were excluded as a competing outcome (Section 2.3).

## 4. Discussion

In this prospective observational study, no statistically significant difference was found between the AUCs of NEWS and qSOFA for hospital admission (0.709 vs. 0.695, *p* = 0.627) or for in-hospital mortality (0.900 vs. 0.862, *p* = 0.150). With 19 deaths, the study was not powered to detect a difference in the size that might be clinically relevant, and the absence of a significant difference is not evidence of equivalence. In the score-specific models, each score was independently associated with both outcomes when modeled with age and comorbid disease in the absence of the other. In a combined exploratory model containing both scores together with supplemental oxygen and GCS, both remained independent predictors of admission (NEWS OR: 1.19, *p* = 0.039; qSOFA OR: 2.67, *p* = 0.013), whereas for mortality only NEWS did (OR: 1.73, *p* = 0.003; qSOFA OR: 1.40, 95% CI: 0.39–4.99, *p* = 0.608). Possible reasons for this loss of significance are considered below.

The sensitivity–specificity trade-off described in the literature provides useful context. In a meta-analysis of 26 studies comprising 62,338 patients with suspected sepsis, Wang et al. reported that qSOFA had the highest specificity but the lowest sensitivity (0.82 and 0.46), whereas NEWS showed intermediate values (0.73 and 0.52); no scoring system achieved both [3]. Our cohort shows the same pattern at the conventional threshold, with a sensitivity of 52.6% and a specificity of 96.8% for mortality at qSOFA ≥ 2. It was only at the lower, cohort-derived qSOFA ≥ 1 threshold that the relationship reversed (sensitivity 84.2%, specificity 80.5%). The profile commonly attributed to a score therefore depends heavily on which threshold is applied, as well as on the score itself and on the population studied, and no single bedside score at a single cutoff is sufficient for mortality prediction in the ED.

Our results are broadly consistent with the larger comparative literature. In 8204 patients with suspected sepsis, Brink et al. found that NEWS outperformed qSOFA and SIRS for 10- and 30-day mortality (AUC: 0.837 vs. 0.744 vs. 0.646) [4], and in 62,403 unselected ED patients, Chen et al. reported NEWS to be significantly more accurate than MEWS and qSOFA across several outcomes, including ED mortality (AUC: 0.862 vs. 0.792) [5]. Similar advantages for NEWS have been reported in immunocompromised sepsis patients [6] and in COVID-19 [7]. Other studies found no statistically significant difference, as we did: Goulden et al. reported similar AUCs in 1818 UK patients with suspected sepsis (0.65 vs. 0.62, *p* = 0.18) [8], and Devia-Jaramillo et al. reported a numerically higher but non-significant AUC for NEWS in 383 Colombian sepsis patients (0.71 vs. 0.68, *p* = 0.293) [9]. The magnitude and statistical significance of any NEWS advantage, therefore, appear to depend on cohort size, case mix, and baseline mortality—factors that plausibly also account for the non-significant difference observed here. Low sensitivity at the conventional qSOFA ≥ 2 threshold is likewise a recurring finding in emergency department cohorts—32.9% in Perman et al. [10] and 31.5% in Prasad et al. [11]—and is consistent with the profile we observed at that threshold.

The behavior of qSOFA across our models merits comment. In the full six-variable model, qSOFA was not an independent predictor of mortality (*p* = 0.608), whereas when modeled without NEWS and without its overlapping components, it was independently associated with mortality (OR 8.30; 95% CI: 3.67–18.78; Firth-corrected 7.40). This estimate is imprecise and should be interpreted together with its confidence interval: only 19 deaths occurred, 17 patients had qSOFA = 2, and one had qSOFA = 3 (Table 11). Moderate collinearity between the scores was confirmed quantitatively (Pearson r = 0.73; VIF 3.19 and 2.64) and may have contributed to the loss of significance in the combined model, although an observational analysis of this size cannot establish that. The defensible conclusion is that our data do not support the claim that qSOFA lacks independent prognostic value and that entering both scores in a single model is methodologically problematic given their shared components.

The cohort-derived qSOFA threshold for mortality (≥1) differs from the conventional ≥2 used in the sepsis literature. A lower threshold is plausible in an etiology-independent high-acuity population, in which even a single positive qSOFA component was associated with a stepwise increase in observed mortality (Table 11). This threshold was, however, both derived and evaluated within the same cohort. Bootstrap evaluation of threshold selection suggested modest optimism (Section 3), but internal validation cannot substitute for external validation, and the correction is itself estimated from the same small sample. This threshold, and the NEWS ≥ 3 admission threshold, are exploratory, cohort-specific observations rather than proposed clinical decision thresholds, and we make no recommendation about which threshold should be used in practice in a population such as this one.

Our findings diverge from those in elderly-specific populations. Sanguanwit et al., studying patients aged ≥60 years with suspected sepsis, found qSOFA ≥ 2 superior to NEWS ≥ 5 for 28-day mortality and septic shock (AUC: 0.66 vs. 0.56, *p* = 0.01) [12]. Given a median age of 66 years in our cohort, this warrants caution when extrapolating to markedly older or comorbidity-heavy subpopulations. A recent prospective Greek evaluation of SIRS, MEWS, NEWS2 and qSOFA in patients with suspected infection similarly found NEWS2 and qSOFA to achieve the most balanced accuracy for 28-day mortality (AUC: 0.77 and 0.79), with mortality remaining elevated in comorbid or elderly patients even when the scores were negative [13]—relevant here, given that 65.9% of our patients had at least one comorbidity. That study used NEWS2 rather than the original NEWS employed here, which should be borne in mind when comparing AUC and cutoff estimates directly.

When admission was disaggregated by level of care (Table 6), the AUC for ICU admission was 0.728 for qSOFA and 0.710 for NEWS, and forward admission, the AUC was 0.607 and 0.638 respectively. Neither difference approached statistical significance (DeLong *p* = 0.634 and 0.393); the event counts were small (35 ICU and 61 ward admissions), and the confidence intervals overlap almost entirely. These are exploratory numerical patterns only and do not support any claim of score-specific strengths according to level of care; testing whether the two scores behave differently across levels of care would require an adequately powered cohort with prespecified, level-of-care-specific outcomes.

Grouped calibration plots and decision curves described the apparent performance of the score-specific models in this cohort (Figure 3 and Figure 4). Calibration assessment was limited by the grouped presentation and, for mortality, by the small number of events. Both models showed positive net benefit relative to treat-all and treat-none strategies across most thresholds examined. These analyses were computed in the development dataset without optimism correction and do not establish performance in other populations or bedside clinical utility. External validation is required.

Finally, the applicability of qSOFA to our population requires explicit framing. The cohorts of Brink et al. [4], Boekhoud et al. [6], Alencar et al. [7], Goulden et al. [8], Devia-Jaramillo et al. [9] and Sanguanwit et al. [12] comprised patients with suspected or confirmed infection—the population for which qSOFA was derived and validated [2]. Only 8.1% of our cohort carried a preliminary diagnosis of sepsis. Two questions must therefore be separated. The first—whether qSOFA performs as intended within its validated indication—cannot be answered here: our subgroup was defined by the preliminary diagnosis of sepsis rather than by suspicion of infection, and with 22 patients and seven deaths, it is uninformative. The second—whether qSOFA carries prognostic information in an undifferentiated high-acuity ED population—is the question this study addresses, and our data are consistent with it doing so, with no significant difference from NEWS. This is a hypothesis for testing in adequately powered cohorts that record suspicion of infection prospectively, not a recommendation to apply qSOFA outside the setting in which it was validated.

One feature of our cohort bears on generalizability. Although all patients were triaged to the high-acuity area, 63.7% were discharged directly home, and the median NEWS was 2 and the median qSOFA was 0. This may reflect the red-area criteria set out in Section 2.2, which are applied at first contact and are designed to capture potential risk of deterioration rather than confirmed critical illness; the category is correspondingly broader than the resuscitation areas of several international triage systems. Most discharged patients had low scores (83.7% with NEWS 0–4; 90.1% with qSOFA 0), although these data cannot verify the appropriateness of individual triage or discharge decisions, and follow-up was limited to our own institution. Our estimates therefore apply to a first-contact high-acuity population with an observed in-hospital mortality of 7.0% and should not be extrapolated to resuscitation-room cohorts with substantially higher baseline severity nor to unselected or low-acuity ED populations.

## 5. Limitations

This study has several limitations. First, it was conducted at a single center, which limits generalizability to emergency departments with different populations and triage practices. Second, and most importantly, only 19 deaths occurred: in the full six-variable mortality model, this yields an events-per-variable ratio of approximately 3.2, far below the recommended minimum of 10. For this reason, the score-specific models (events-per-variable 6.3, still modest) rather than the full model are used as the basis for interpretation; Firth’s penalized regression was applied as a sensitivity analysis, and all mortality analyses are reported as exploratory; the wide confidence interval for qSOFA in Model B (3.67–18.78) illustrates the imprecision directly. Third, the score-specific models, the outcome hierarchy used for reporting, and the sepsis subgroup analysis were decided during revision rather than in advance, and covariates were selected on clinical grounds rather than by a formal algorithm; these choices are transparent but not prespecified. Fourth, the optimal cutoffs were derived by the Youden index within the same dataset in which they were evaluated, and the calibration plots and decision-curve analysis were likewise computed in the development dataset without optimism correction; none of these can substitute for external validation. Fifth, scores were calculated only at ED presentation; serial measurement, which improves prognostic accuracy [14], was not performed. Sixth, no a priori sample-size calculation was performed, and the study may be underpowered to detect true differences between the scores. Seventh, the subgroup analysis was defined by the preliminary diagnosis of sepsis rather than by suspicion of infection, comprised only 22 patients with seven deaths, and cannot assess qSOFA within its validated indication. Eighth, outcome ascertainment was confined to our own institution, and no systematic search of national death records was performed, so deaths, readmissions or ED presentations elsewhere were not systematically sought, and post-discharge adverse events are a minimum estimate. Finally, only patients triaged to the high-acuity area were included, so performance in lower-acuity or infection-specific ED populations could not be assessed.

## 6. Conclusions

In this prospective single-center study of 270 high-acuity emergency department patients, no statistically significant difference was found between the AUCs of NEWS and qSOFA for hospital admission (0.709 vs. 0.695) or in-hospital mortality (0.900 vs. 0.862). Each score was independently associated with both outcomes when modeled separately from the other, and the between-score differences in AUC for ICU and ward admission were not statistically significant. The cohort was not selected for suspected infection, so these findings do not speak to the performance of qSOFA within its validated indication. The study is exploratory and hypothesis-generating rather than confirmatory: with 19 deaths, the mortality analyses are underpowered, the cohort-derived thresholds are not externally validated, and the calibration and decision-curve analyses were computed in the development dataset. We therefore draw no recommendation for one score over the other, and none for clinical use of the thresholds reported here. Larger, adequately powered multicenter studies with external validation are needed before these findings can inform practice.

## Figures and Tables

**Figure 1:**
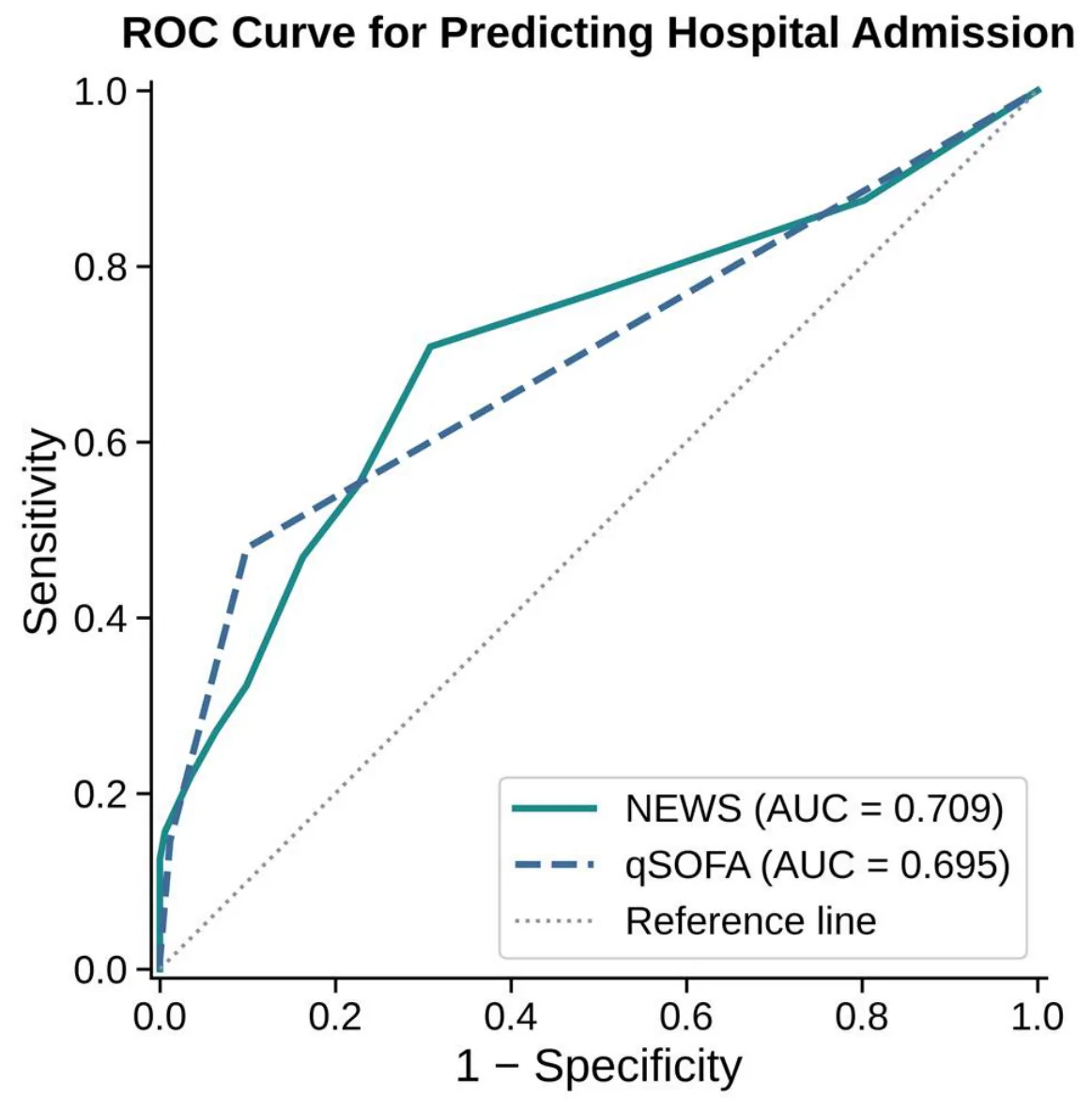
ROC curve for NEWS and qSOFA in predicting hospital admission.

**Figure 2:**
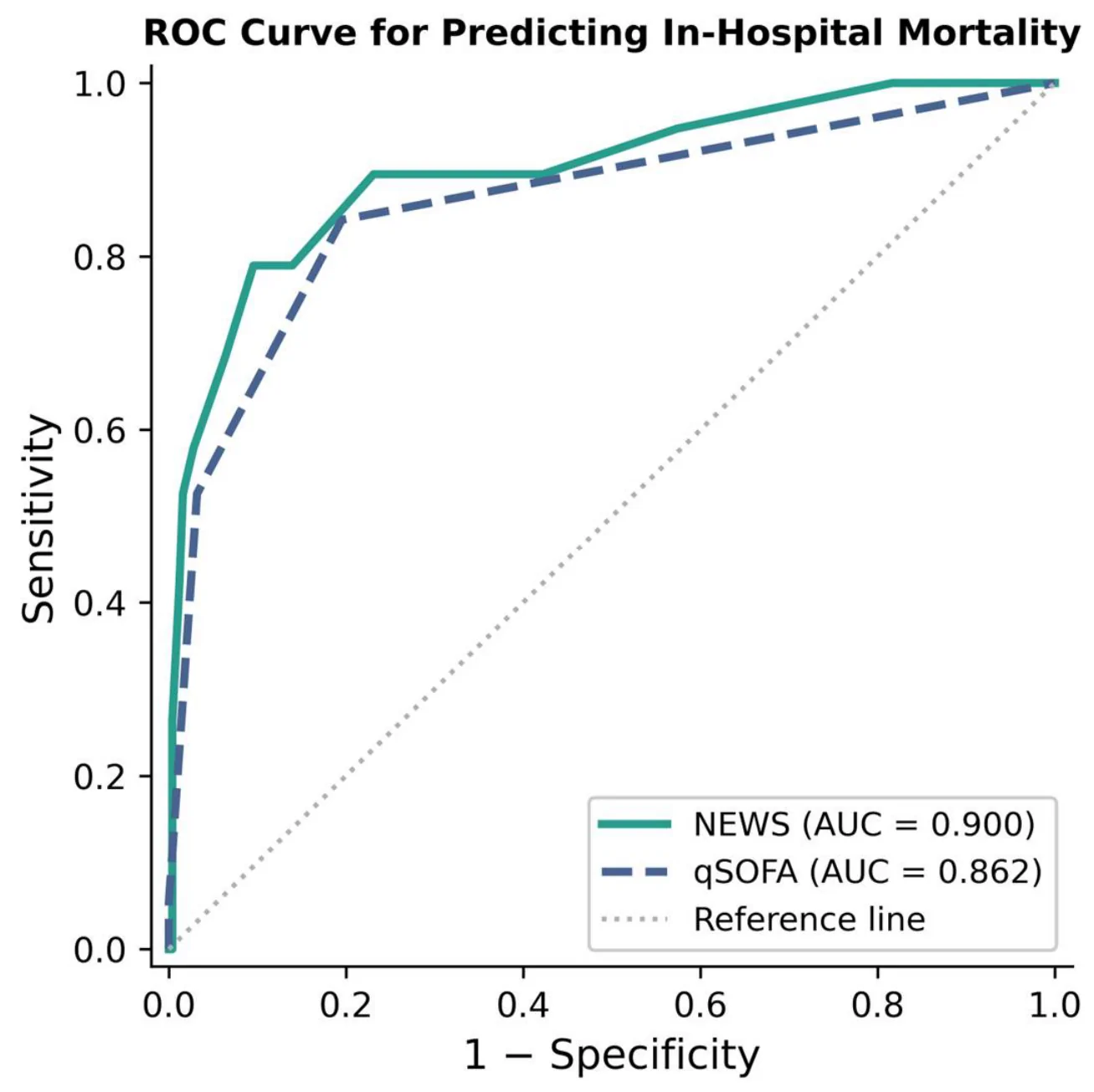
ROC curve for NEWS and qSOFA in predicting in-hospital mortality.

**Figure 3:**
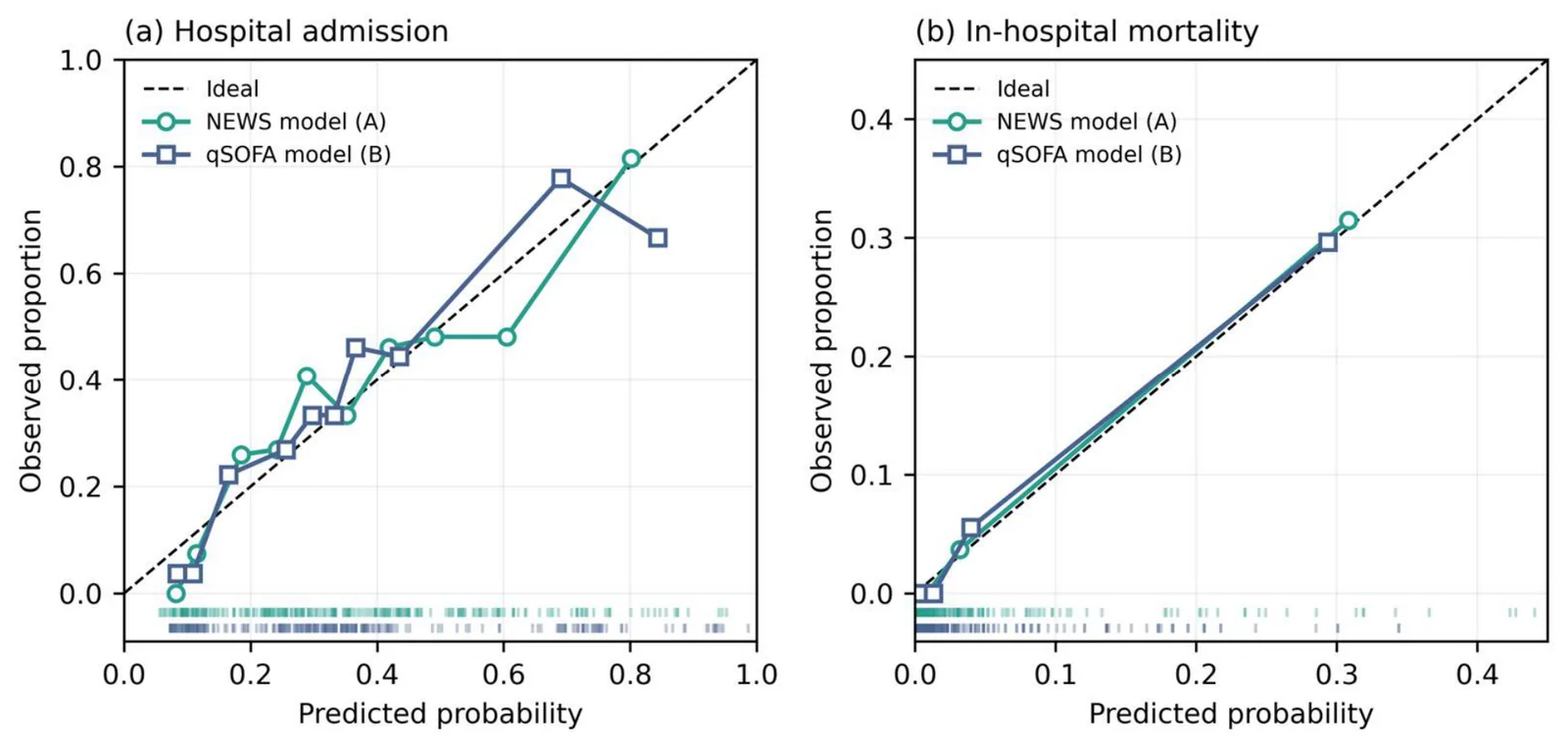
Calibration of the score-specific models: observed versus predicted probability for (**a**) hospital admission (*n* = 268, 96 events; deciles of predicted risk) and (**b**) in-hospital mortality (*n* = 270, 19 events; quintiles). Model A = NEWS with age and comorbid disease; Model B = qSOFA with age and comorbid disease. Predicted probabilities are from the standard (unpenalized) logistic models. These are apparent calibration plots computed in the development dataset and are not optimism-corrected;. Tick marks show the distribution of predicted probabilities.

**Figure 4:**
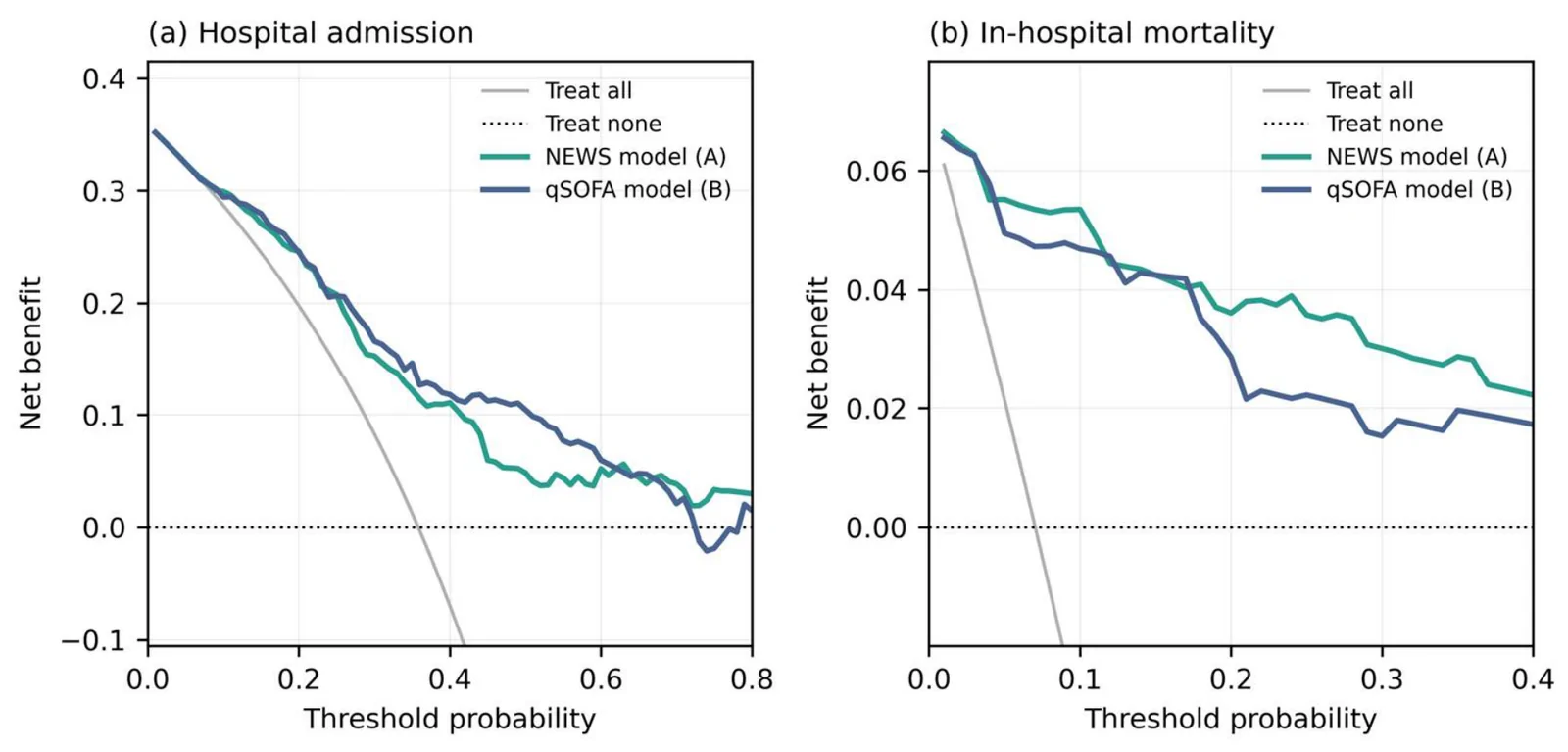
Decision-curve analysis showing the net benefit of the NEWS-based (Model A) and qSOFA-based (Model B) models relative to treat-all and treat-none strategies for (**a**) hospital admission (*n* = 268, 96 events) and (**b**) in-hospital mortality (*n* = 270, 19 events). Both models include age and comorbid disease alongside the score. Curves were computed in the development dataset from the standard (unpenalized) logistic models and are not optimism-corrected.

**Table 1:** Demographic and clinical characteristics.

Variable	Value
Age, years, median (IQR)	66.0 (47.3–77.0)
Female, *n* (%)	129 (47.8)
Male, *n* (%)	141 (52.2)
Comorbidity present, *n* (%)	178 (65.9)
Supplemental O_2_, *n* (%)	59 (21.9)
GCS, median (IQR)	15 (15–15)
Pulse rate, beats/min, median (IQR)	90 (76.3–106)
Respiratory rate, breaths/min, median (IQR)	18 (17–20)
SBP, mmHg, median (IQR)	130 (110–156)
DBP, mmHg, median (IQR)	75 (64–86)
Mean arterial pressure, mmHg, mean ± SD	96.0 ± 20.5
Saturation, %, median (IQR)	97 (94–98)
Temperature, °C, median (IQR)	36.4 (36.2–36.7)
NEWS, median (IQR)	2 (1–5)
qSOFA, median (IQR)	0 (0–0)
Time to death, mean ± SD (days; *n* = 18)	4.61 ± 3.52

Continuous variables are presented as median (IQR) unless otherwise stated.

**Table 2:** Comparison of discharged and admitted patients (*n* = 268; two ed deaths excluded).

Variable	Discharged (*n* = 172)	Admitted to Ward/ICU (*n* = 96)	*p*-Value
Age	57.5 (41–73)	73.0 (61–81)	<0.001
Comorbidity present, *n* (%)	97 (56.4)	79 (82.3)	<0.001
Supplemental O_2_, *n* (%)	23 (13.4)	34 (35.4)	<0.001
GCS	15.0 (15–15)	15.0 (15–15)	<0.001
NEWS	1.5 (1–3)	4.0 (2–7)	<0.001
qSOFA	0 (0–0)	0 (0–1)	<0.001

Continuous variables are presented as median (IQR) and compared with the Mann–Whitney U test; categorical variables are presented as *n* (%) and compared with the chi-square test with Yates’ continuity correction.

**Table 3:** Comparison by in-hospital mortality status.

Variable	Survivors (*n* = 251)	Non-Survivors (*n* = 19)	*p*-Value
Age	64.0 (45.5–75.0)	82.0 (75.0–84.5)	<0.001
Comorbidity present, *n* (%)	162 (64.5)	16 (84.2)	0.135
Supplemental O_2_, *n* (%)	45 (17.9)	14 (73.7)	<0.001
GCS	15.0 (15.0–15.0)	14.0 (10.5–15.0)	<0.001
NEWS	2.0 (1.0–4.0)	10.0 (7.0–11.5)	<0.001
qSOFA	0 (0–0)	2.0 (1.0–2.0)	<0.001

Continuous variables are presented as median (IQR) and compared with the Mann–Whitney U test; categorical variables are presented as *n* (%) and compared with the chi-square test with Yates’ continuity correction.

**Table 4:** ROC analysis results.

Score	AUC	95% CI	*p*-Value	DeLong *p* (NEWS vs. qSOFA)
NEWS (Admission)	0.709	0.641–0.777	<0.001	0.627
qSOFA (Admission)	0.695	0.639–0.750	<0.001	
NEWS (Mortality)	0.900	0.814–0.985	<0.001	0.150
qSOFA (Mortality)	0.862	0.767–0.958	<0.001	

Admission analyses are based on *n* = 268; the two patients who died in the ED were excluded as a competing outcome (Section 2.3).

**Table 5:** Predictive values and likelihood ratios at data-derived and conventional cutoffs.

Outcome	Score (Cutoff)	Sensitivity	Specificity	PPV	NPV	LR+	LR−
Admission	NEWS (≥3) a	70.8%	69.2%	56.2%	81.0%	2.30	0.42
Admission	NEWS (≥5) b	46.9%	83.7%	61.6%	73.8%	2.88	0.64
Admission	NEWS (≥7) b	27.1%	93.6%	70.3%	69.7%	4.24	0.78
Admission	qSOFA (≥1) a	47.9%	90.1%	73.0%	75.6%	4.85	0.58
Admission	qSOFA (≥2) b	14.6%	98.8%	87.5%	67.5%	12.54	0.86
Mortality	NEWS (≥5) b	89.5%	76.9%	22.7%	99.0%	3.87	0.14
Mortality	NEWS (≥7) a,b	78.9%	90.4%	38.5%	98.3%	8.26	0.23
Mortality	qSOFA (≥1) a	84.2%	80.5%	24.6%	98.5%	4.31	0.20
Mortality	qSOFA (≥2) b	52.6%	96.8%	55.6%	96.4%	16.51	0.49

a Data-derived (Youden index) cutoff. b Conventional cutoff used in routine practice and in most comparative studies. Admission analyses are based on *n* = 268; the two patients who died in the ED were excluded as a competing outcome (Section 2.3).

**Table 6:** AUC for composite admission, ICU admission, and ward admission (secondary analysis).

Outcome	Events/Total, *n*/*N*	NEWS AUC (95% CI)	qSOFA AUC (95% CI)	DeLong *p*
Hospital admission (composite)	96/268	0.709 (0.641–0.777)	0.695 (0.639–0.750)	0.627
ICU admission only	35/268	0.710 (0.604–0.816)	0.728 (0.638–0.819)	0.634
Ward admission only	61/268	0.638 (0.558–0.718)	0.607 (0.540–0.674)	0.393

Events/total: for ICU admission, the comparison group comprises all other evaluable patients, including those admitted to the ward; for ward admission, it comprises all other evaluable patients, including those admitted to the ICU. Admission analyses are based on *n* = 268; the two patients who died in the ED were excluded as a competing outcome (Section 2.3).

**Table 7:** Logistic regression analysis predicting hospital admission (full six-variable model; exploratory).

Variable	OR	95% CI	*p*-Value
NEWS	1.19	1.01–1.41	0.039
qSOFA	2.67	1.23–5.77	0.013
Comorbid disease	2.45	1.12–5.32	0.024
Age	1.02	1.00–1.04	0.087
Supplemental O_2_	0.57	0.22–1.51	0.261
GCS	0.72	0.45–1.15	0.173

Odds ratios are per 1-point increase for NEWS, qSOFA and GCS and per 1 year for age; for comorbid disease and supplemental oxygen, the reference category is the absence of the characteristic. Admission analyses are based on *n* = 268; the two patients who died in the ED were excluded as a competing outcome (Section 2.3).

**Table 8:** Score-specific models predicting hospital admission.

Model	Variable	OR	95% CI	*p*-Value
Model A	NEWS	1.29	1.17–1.43	<0.001
(AIC = 298.8)	Age	1.02	1.001–1.038	0.036
	Comorbid disease	2.04	0.996–4.195	0.051
Model B	qSOFA	4.85	2.74–8.58	<0.001
(AIC = 288.7)	Age	1.02	1.001–1.039	0.039
	Comorbid disease	2.29	1.08–4.83	0.030

Odds ratios are per 1-point increase in NEWS or qSOFA and per 1 year for age; the reference category for comorbid disease is absence of comorbidity. Admission analyses are based on *n* = 268; the two patients who died in the ED were excluded as a competing outcome (Section 2.3).

**Table 9:** Logistic regression analysis predicting mortality (full six-variable model; exploratory).

Variable	OR	95% CI	*p*-Value
NEWS	1.73	1.21–2.47	0.003
qSOFA	1.40	0.39–4.99	0.608
Age	1.10	1.03–1.18	0.005
Comorbid disease	0.48	0.10–2.37	0.368
Supplemental O_2_	0.22	0.03–1.79	0.157
GCS	0.69	0.48–0.98	0.040

Odds ratios are per 1-point increase for NEWS, qSOFA, and GCS and per 1 year for age; for comorbid disease and supplemental oxygen, the reference category is absence of the characteristic.

**Table 10:** Score-specific models predicting in-hospital mortality with Firth sensitivity analysis (exploratory).

Model	Variable	Method	OR	95% CI	*p*-Value
Model A	NEWS	Standard	1.65	1.36–2.01	<0.001
(AIC = 81.5)	NEWS	Firth	1.60	1.34–1.91	<0.001
	Age	Standard	1.08	1.02–1.15	0.006
	Age	Firth	1.08	1.02–1.13	0.006
	Comorbid disease	Standard	0.56	0.12–2.57	0.456
	Comorbid disease	Firth	0.53	0.13–2.23	0.386
Model B	qSOFA	Standard	8.30	3.67–18.78	<0.001
(AIC = 87.8)	qSOFA	Firth	7.40	3.44–15.89	<0.001
	Age	Standard	1.07	1.02–1.13	0.006
	Age	Firth	1.07	1.02–1.12	0.006
	Comorbid disease	Standard	0.86	0.19–3.94	0.843
	Comorbid disease	Firth	0.78	0.19–3.24	0.728
Full model (Firth) †	NEWS	Firth	1.60	1.16–2.20	0.004
	qSOFA	Firth	1.42	0.43–4.65	0.564
	Age	Firth	1.09	1.03–1.15	0.005
	Comorbid disease	Firth	0.47	0.11–2.02	0.312
	Supplemental O_2_	Firth	0.29	0.04–1.88	0.193
	GCS	Firth	0.73	0.52–1.01	0.057

Odds ratios are per 1-point increase for NEWS, qSOFA, and GCS and per 1 year for age; for comorbid disease and supplemental oxygen, the reference category is absence of the characteristic. AIC values shown beside Model A (81.5) and Model B (87.8), and † AIC = 77.7 for the full model, all refer to the standard (non-Firth) models.

**Table 11:** Distribution of qSOFA values and observed in-hospital mortality.

qSOFA	*N*	Deaths	In-Hospital Mortality (%)
0	205	3	1.5%
1	47	6	12.8%
2	17	9	52.9%
3	1	1	100.0%

**Table 12:** Internal validation of the score-specific models (2000 bootstrap resamples).

Outcome	Model	AUC (Apparent)	AUC (Corrected)	Brier (Apparent)	Brier (Corrected)	Calibration Slope
Admission	Model A (NEWS)	0.758	0.748	0.186	0.192	0.95
Admission	Model B (qSOFA)	0.781	0.774	0.177	0.182	0.96
Mortality	Model A (NEWS)	0.950	0.936	0.041	0.045	0.88
Mortality	Model B (qSOFA)	0.936	0.920	0.044	0.048	0.87

Corrected values are optimism-corrected by bootstrap resampling (Harrell). Model A = score + age + comorbid disease with NEWS; Model B = the same with qSOFA. Admission analyses are based on *n* = 268 (Section 2.3).

## Data Availability

The data presented in this study are available on request from the corresponding author, due to patient privacy and institutional data protection restrictions.
